# Is ^1^H-MR spectroscopy useful as a diagnostic aid in MSA-C?

**DOI:** 10.1186/s40673-019-0099-0

**Published:** 2019-07-05

**Authors:** Viren H. Kadodwala, Marios Hadjivassiliou, Stuart Currie, Nicholas Skipper, Nigel Hoggard

**Affiliations:** Academic Unit of Radiology, C Floor, Royal Hallamshire Hospital, Glossop Road, Sheffield, S10 2JF UK

**Keywords:** MSA, MRS, Multiple system atrophy, MR spectroscopy, Ataxia

## Abstract

**Background:**

Multiple system atrophy (MSA) is a sporadic adult-onset neurodegenerative disease with a cerebellar subtype where ataxic symptoms predominate (MSA-C) associated with autonomic dysfunction and a grave prognosis. The purpose of this analysis was to identify if cerebellar volumetry and MR spectroscopy obtained as part of routine clinical work up of patients with sporadic ataxia differentiates patients with multiple system atrophy- cerebellar type (MSA-C) from those with sporadic adult-onset ataxia of unknown etiology (SAOA) who’s condition follows a more benign course.

**Methods:**

Retrospective comparison was undertaken of 20 clinically probable or possible MSA-C patients, 20 age and sex matched patients with SAOA and 20 healthy control subjects. Single voxel ^1^H-MR spectroscopy of the cerebellar hemisphere and vermis and volumetric analysis of the cerebellum and brainstem were undertaken on baseline scans, comparing all groups.

**Results:**

There was significant reduction in NAA/Cr levels in patients with MSA-C when compared to those with ISA (*p* = 0.005) and healthy controls (*p* < 0.001) in both the hemisphere and vermis. Brainstem volume was significantly reduced in MSA-C patients compared to SAOA patients (*p* < 0.001) and healthy controls (*p* < 0.001). There was no difference in cerebellar volume between MSA-C patients and SAOA patients.

**Conclusion:**

This paper demonstrates that at presentation, MSA-C patients have a significant reduction of NAA/Cr in the cerebellum and significant decrease in brainstem volume when compared to SAOA and healthy controls. This is the first study to sucessfully show clinical utility of MR spectroscopy of the cerebellum for differentiating MSA-C from patients with SAOA.

**Electronic supplementary material:**

The online version of this article (10.1186/s40673-019-0099-0) contains supplementary material, which is available to authorized users.

## Introduction

Multiple system atrophy (MSA) is a sporadic adult-onset neurodegenerative disease with a cerebellar subtype where ataxic symptoms predominate (MSA-C). In addition to the motor symptoms MSA-C patients develop severe autonomic failure manifesting as urinary dysfunction, erectile dysfunction and orthostatic hypotension [1].

The prognosis of MSA-C patients is poor; with a median survival time of around eight years [2], hence the importance of differentiating patients with MSA-C from other cerebellar ataxias [3].

The diagnosis of MSA is only definitive at post mortem with the demonstration of an α-synucleinopathy. In life the diagnosis is termed either probable or possible depending on clinical findings, as set out in Additional file 1 modified from the second consensus statement on the diagnosis of MSA [1]*.* Sporadic adult-onset ataxia of unknown etiology (SAOA) is not a diagnosis as such, but represents a common situation faced by neurologists in ataxia clinics. The diagnosis of SAOA, by definition, is based entirely on the diagnostic elimination of all other causes of ataxia (genetic or acquired) [4]. The clinical distinction between MSA-C and SAOA is difficult at the early stages of the disease [4–6]. Thus although patients with MSA-C may be included amongst those with a positive diagnosis for their ataxia and are labelled with SAOA, as time passes do become clinically distinct. Yet such a diagnosis is extremely important given that the prognosis of SAOA is much more benign when compared to MSA-C and potential treatments for MSA-C would be best instituted as early as possible given their relatively rapid progression [3, 7].

The purpose of this study was to evaluate if analysis of cerebellar atrophy and MR spectroscopy obtained as part of routine clinical work up of patients with ataxia referred to the Sheffield Ataxia Centre could differentiate patients with MSA-C from those with SAOA. The use of MR spectroscopy for assessment of ataxias has been previously described and summarised by Baldarçara et al. and its clinical application by Hadjivassiliou et al. [8, 9].

## Methods

### Patients and controls

A retrospective analysis of the first imaging from patients attending the Sheffield Ataxia Centre was performed. The diagnosis of MSA-C was based on the clinical history in accordance to the published guidelines [1] and neuroimaging findings. SAOA patients were diagnosed by exclusion of identifiable causes. A full history and examination was conducted. Extensive investigations to determine the aetiology of ataxia were performed. Such tests, depending on clinical indications, included blood cell counts, biochemistry, thyroid function, serology for coeliac disease, vitamin A, B12, E; metabolic screen for acyl-carnitines, urinary organic acids, very long chain fatty acids, phytanic acids, lysosymal enzymes, lipoproteins, serum electrophoresis, copper, caeruloplasmin, ferritin and iron, common mitochondrial genetics (MELAS, MERRF, NARP) and in selected cases when clinically indicated, mutations in the mitochondrial DNA polymerase gamma (*POLG*) gene as well as muscle biopsies. In addition, testing to exclude other ataxia syndromes was performed, including spinocerebellar ataxias (SCA1, 2, 3, 6, 7, 12 and 17) and Friedreich’s ataxia (FA). Additional genetic tests including, for example, *DRPLA*, fragile X, ataxia occulomotor apraxia, episodic ataxias, SPG7 screening were carried out if clinically indicated. The introduction of next generation sequencing meant that all the ataxia patients in this study underwent further genetic testing on an ataxia gene panel (42 genes) and none were positive.

Patients were excluded if they had identifiable causes of ataxia. The SAOA patients were matched to MSA-C patients for age at first scan. The clinical records were assessed to obtain the clinical symptoms of the patients, the duration of ataxia and severity of the ataxia. The severity of ataxia was divided into three categories by an experienced consultant neurologist as published previously [10, 11]: mild (instability without staggering steps or falls), moderate (instability with staggering steps or falls and/or needs walking aid), and severe (unable to walk despite support from accompanying person).

The MSA-C patients were matched to healthy controls for age at their scan and sex. The healthy controls underwent a thorough screening health questionnaire to ensure they had no form of illness that could result in cerebellar dysfunction.

The project had IRB approval to use patient’s clinical data without written consent. Fully informed written consent was obtained for all healthy control subjects.

### Imaging protocol

Scanning was performed using a 3 T system (Philips ACHIEVA 3.0 T Best, Netherlands) with an 8-channel receive only array head coil. The imaging data analysed from patients was from their initial MR scan with us that included our routine imaging protocol for patients with ataxia. The imaging protocol was as follows:

#### ^1^H-MR spectroscopy

**Fig. 1:**
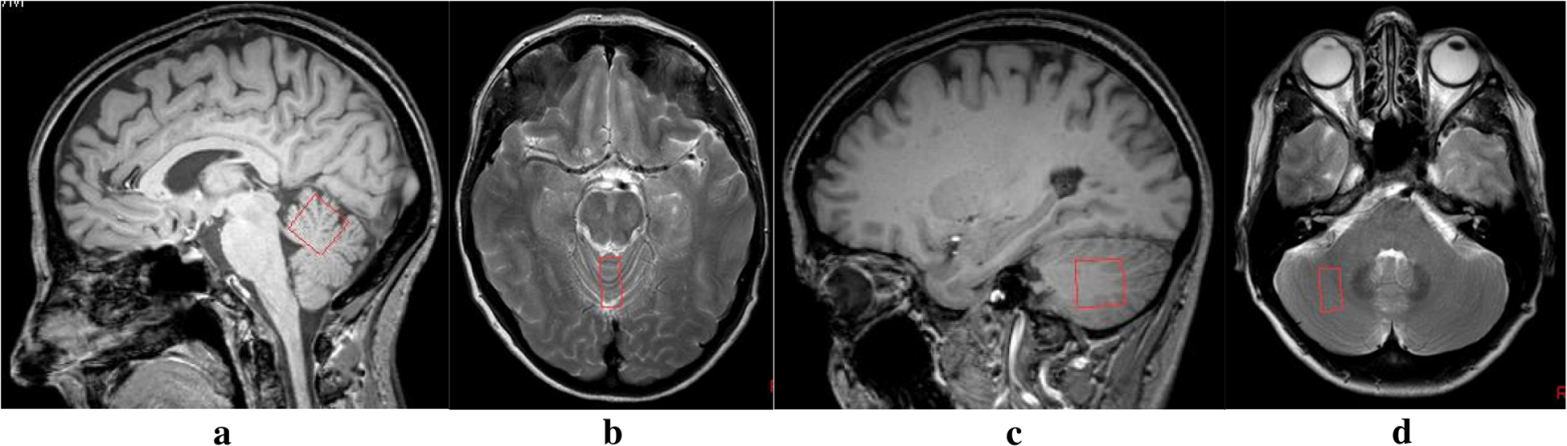
shows example localising MR images demonstrating the placement of the voxels of interest, both measuring 2 cm by 2 cm by 1 cm, for MR spectroscopy of cerebellum performed on all patients. **a** Sagittal T1 image [far left] and **b** axial T2 weighted image [centre left] showing the voxel of interest for the vermis. **c** Sagittal T1 image [centre right] and **d** Axial T2 weighted image [far right] showing the voxel of interest for the right cerebellar hemisphere

A point-resolved spectroscopy (PRESS) sequence (TR = 2000 ms, TE = 144 ms; 128 measurements; 1024 spectral points; spectral bandwidth 2000 Hz) acquired data at two voxel positions, the superior cerebellar vermis and the deep cerebellar white matter of the right hemisphere, avoiding the dentate nucleus that represents a separate spectroscopy target in our experience. The voxel size was 2.0 × 1.0 × 2.0 cm^3^. Careful placement of the voxel ensured cerebrospinal fluid contamination of the voxel was minimised. Chemical shift selective imaging pulse technique (CHESS) was used for water suppression. Post processing of the spectra involved zero filling, Gaussian filtering, exponential multiplication, Fourier transform and manual phase correction with baseline subtraction. The quality of the spectra was assessed by two neuroradiologists to ensure adequacy. Figure 1 shows the placement of the voxel in both the cerebellar vermis and hemisphere.

#### Structural MR imaging

High-resolution three dimensional T1-weighted MR imaging scans were acquired using a magnetisation prepared rapid gradient echo sequence with TR = 11 ms, TE = 4.8 ms, flip angle = 8^o^, field of view = 256 × 205 × 150 mm, voxel dimension = 0.8 mm (isotropic). Axial T2-weighted images were acquired using a turbo spin echo sequence (TR = 3000 ms, TE = 80 ms; echo train length = 14; 1 NSA, voxel dimension = 0.45 × 0.44 × 4 mm).

#### Volumetric analysis

FMRIB’s Software Library (FSL) [12–16] was used to perform the structural image volume analysis. The software automatically quantified the volume of the cerebellum and the brainstem and fourth ventricle. Structural Image Evaluation using Normalization of Atrophy cross-sectional method (SIENAX) [17] was used to calculate the brain tissue volume, correcting for subject head size.

The volume of the brainstem and fourth ventricle is calculated as one entity using FSL [18]. The avoid effects of change in the fourth ventricle it was manually segmented on each MR image it was shown on to quantify the volume blinded to whether the images were from a control participant or patient using an extended workstation Philips Medical Systems. The brainstem boundaries were the foramen magnum inferiorly and superiorly by a line drawn across the superior aspect of the midbrain at the level of its interface as the floor of the third ventricle.

#### Imaging outcomes measures

The following outcomes were measured: (1) brainstem volume expressed as a percentage of total intracranial volume; (2) cerebellar volume expressed as a percentage of total intracranial volume; (3) NAA/Cr and Cho/Cr ratios in the cerebellar hemisphere and vermis. Additionally the presence of the hot cross bun sign was noted.

### Statistics analysis

Statistical analysis was performed using Statistical Package for Social Sciences (SPSS) version 20 and MedCalc version 12.3. One way ANOVA was used to compare MSA-C patients with controls and SAOA patients. Post hoc testing was performed by Tukey’s HSD if a significant difference (*p* < 0.05) was elicited by ANOVA across the study data.

## Results

The patient demographics are shown in Table 1. In total 60 study subjects were used, split into three groups. There was no significant difference between the age at scan of patients with MSA-C (58.7 years), SAOA (58.2 years) and controls subjects (57.9 years) [ANOVA (*p* > 0.05)]. The sex of subjects was also matched between groups.

**Table 1:** Patient demographics

	MSA-C *N* = 20	SAOA *N* = 20	Healthy controls *N* = 20
Male	14	14	13
Age (mean years (SD))	58.2 (6.6)	58.2 (7.2)	58.2 (6.5)
Mean severity of ataxia (SD)	2.8 (0.60)	1.38 (0.59)	NA
Mean duration of symptoms (years (SD))	2.80 (1.65)	8.2 (6.12)	NA

Group demographics

Age and severity and duration of symptoms are in relation to the acquisition of MR imaging

There was no significant difference between the age of MSA-C, SAOA and control subjects [ANOVA (*p* > 0.05)]

The mean value of ataxia severity was significantly greater in MSA-C patients compared to SAOA [ANOVA and Tukey’s post hoc analysis (*p* < 0.001)]

The clinical features of the patients with MSA-C and SAOA were retrospectively obtained from the patients’ clinical notes (see Table 2). All patients presented with ataxia. All patients with MSA-C had in addition either urinary symptoms or impotence and 16 of the 20 had either symptomatic or asymptomatic orthostatic hypotension. On subsequent follow-up *all* patients labelled with MSA-C in this study had developed the clinical features that permitted the diagnosis of probable MSA-C. Specifically, long-term follow up was available in 17 patients of the MSA-C group. Three could no longer attend due to the travelling distances involved and their severe disability. Of the 17 MSA-C patients with long term follow up data, all are now dead with a mean age at death of 62.5 years (range 48 to 75 years). In the SAOA cohort long term follow up was available in 18 out of the 20 patients; there have been 5 deaths by contrast to 17 in the MSA-C, (mean age 69.7 years).

**Table 2:** Frequency of symptoms in MSA-C patients at presentation and first scan

Symptom		Presentation	First Scan
Number of patients		20	20
Urinary	Frequency	9 (45)^a^	11 (55)
	Urgency	13 (65)	13 (65)
	Incontinence	9 (45)	9 (45)
	Nocturia	9 (45)	11 (55)
	Bladder dysfunction	19 (95)	19 (95)
Orthostatic Hypotension	BP fall	15 (75)	15 (75)
	Significant BP	13 (65)	13 (65)
	Symptomatic	5 (25)	8 (40)
Impotence		11 (78.6)^b^	11 (78.6)^b^
Cerebellar Syndrome	Gait ataxia	20 (100)	20 (100)
	Limb ataxia	20 (100)	20 (100)
	Dysarthria	17 (85)	17 (85)
	Nystagmus	10 (50)	10 (50)
	Cerebellar dysfunction	20 (100)	20 (100)
Parkinsonism	Tremor	0 (0.0)	0 (0.0)
	Rigidity	3 (15)	3 (15)
Other	Vivid dreams	6 (30)	6 (30)
	Hyper-reflexia	7 (35)	7 (35)

^a^Number of MSA-C subjects with symptom (% of total number of MSA-C subjects with symptom)

^b^11 of the 14 male subjects presented with impotence

The mean value of ataxia severity was significantly greater in MSA-C patients compared to both SAOA [ANOVA and Tukey’s post hoc analysis (*p* < 0.001)]. See Table 3.

**Table 3:** Summaries the ataxia severity grading across the 2 patient groups in the study

Ataxia severity (number of patients)	MSA-C	SAOA
Mild	0	13
Moderate	7	6
Severe	13	1
Mean value for ataxia severity	2.6 (0.49)	1.4 (0.6)

### Imaging results

#### ^1^H-MR spectroscopy results

MR spectroscopy results are summarised in Table 4.

**Table 4:** Summary of imaging results across the 2 groups of ataxic patients (MSA-C, SAOA) and healthy control subjects. One-way ANOVA did not demonstrate a significant difference in Cho/Cr from the hemisphere, so post hoc testing was not preformed

	MSA-C		SAOA		MSA-C vs SAOA	Controls		MSA-C compared to controls
	Mean	SD	Mean	SD	*P*-value	Mean	SD	*P*-value
Vermis NAA/Cr	0.67	0.12	0.82	.16	P = 0.003‡	0.99	0.10	*P* < 0.001^+‡^
Vermis Cho/Cr	0.62	0.25	0.76	.18	*P* > 0.05	0.80	0.08	*P* = 0.040^+^
Hemisphere NAA/Cr	0.72	0.25	0.97	.20	*P* = 0.001‡	1.01	0.09	*P* < 0.001^+‡^
Hemisphere Cho/Cr	0.70	0.21	0.82	.22	*P* > 0.05	0.78	0.12	*P* > 0.05
Brainstem volume (% of TICV)	0.72	0.22	1.16	.18	P < 0.001‡	1.25	0.19	*P* < 0.001^+‡^
Cerebellum volume (% of TICV)	5.64	0.60	4.97	1.12	*P* > 0.05	7.53	1.15	*P* < 0.001^+‡^
Brainstem volume (cm^3^)	10.86	3.33	17.02	2.49	*P* < 0.001^+‡^	18.95	2.64	*P* < 0.001^+‡^
Cerebellum volume (cm^3^)	82.17	8.16	72.87	16.29	*P* = 0.078+	115.28	15.14	*P* < 0.001^‡^

+ANOVA with Tukey’s HSD post hoc

‡remain statistically significant after Bonferoni correction

##### Patients with MSA-C compared to healthy control subjects

**Fig. 2:**
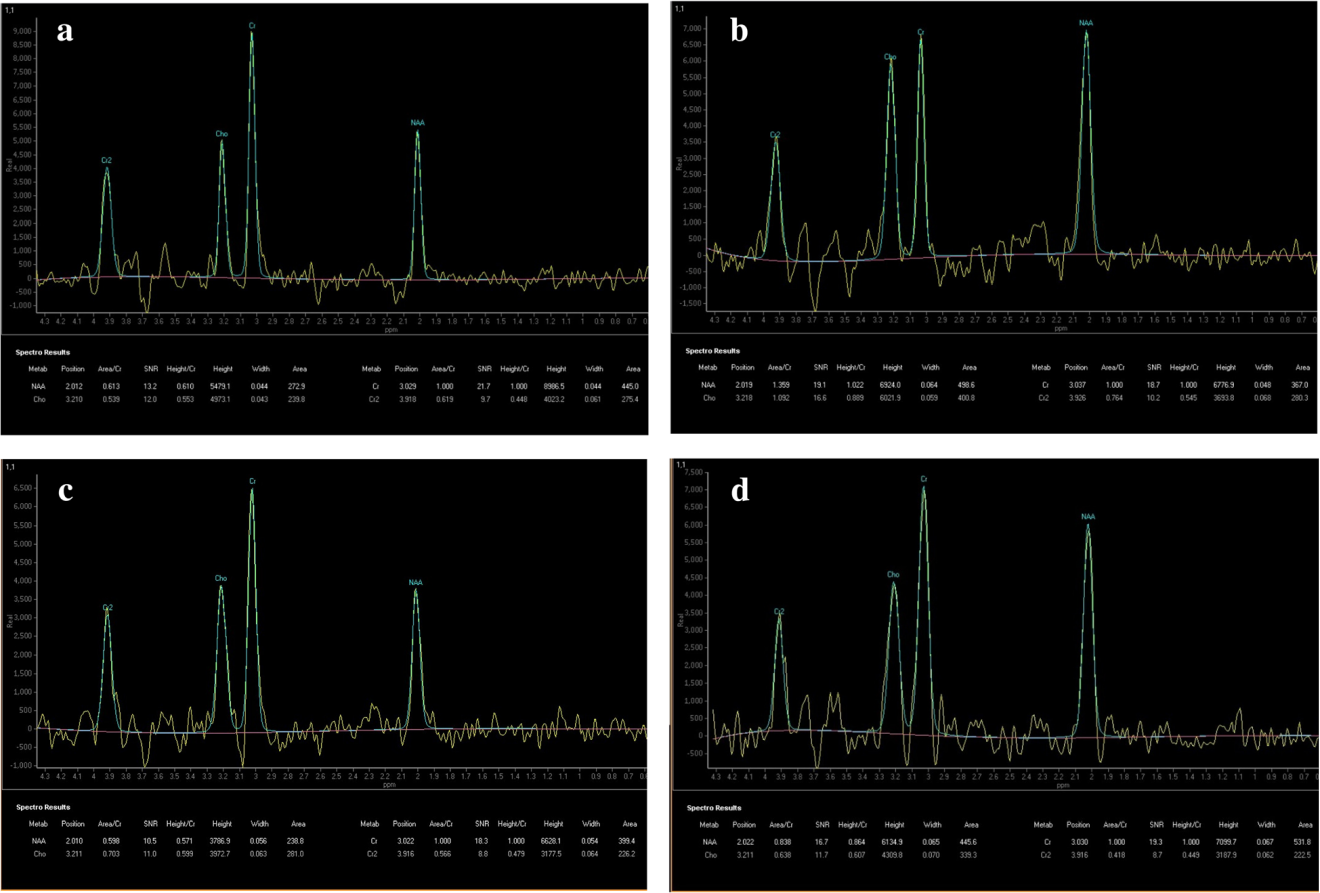
shows the MR spectra that were obtained from a 67 years old female with a subsequent clinical diagnosis of probable MSA-C and the spectroscopy from a follow up study. These spectra show the initially markedly reduced NAA level within the vermis relative to the degree of atrophy and normal values returned from the hemisphere that contrast with the rapidly declining levels from the hemisphere at follow up one year later. There was a subtle hot cross bun sign visible on the follow up cross sectional T2 weighted imaging at the time of the follow up imaging and spectroscopy. **a** and **b** are the spectra obtained at presentation from the vermis and right cerebellar hemisphere respectively. **c** and **d** are the spectra obtained at follow up one year later from the vermis and right cerebellar hemisphere respectively

NAA/Cr (0.67 ± 0.12) and Cho/Cr (0.62 ± 0.25) from the vermis were significantly reduced in patients with MSA-C when compared to age- and sex-matched controls [NAA/Cr = 0.99 ± 0.10, p < 0.001, Cho/Cr = 0.80 ± 0.08,, *p* = 0.04]. NAA/Cr (0.72 ± 0.25) from the cerebellar hemisphere in patients with MSA-C was significantly reduced compared to healthy control subjects [1.01 ± 0.09 p < 0.001]. There was no significant difference in hemispheric Cho/Cr of patients with MSA-C compared to controls. Figure 2 shows the MR spectra obtained from a patient at presentation and then at one year follow up subsequently clinically diagnosed with probable MSA-C. This example shows the relatively marked severity and rapid decline of spectroscopic changes in the cerebellum of a patient with clinically probable MSA-C.

##### Patients with MSA-C compared to patients with SAOA

Vermian NAA/Cr was significantly reduced in patients with MSA-C (0.67 ± 0.12) compared to patients with SAOA (0.82 ± 0.16), *p* = 0.003. Cho/Cr from the vermis was not significantly different between patients with MSA-C and SAOA.

NAA/Cr in the hemisphere was significantly reduced in patients with MSA-C compared to patients with SAOA (0.672 ± 0.25) versus (0.97 ± 0.20), *p* = 0.001. Cho/Cr from the hemisphere was not significantly different between patients with MSA-C and SAOA.

#### Hot cross bun sign and volumetric analysis

Fifteen patients with clinically probable MSA-C were shown to have the hot cross bun sign but five did not display the sign at the time of the initial scan at presentation. None of the patients with SAOA or control subjects had a hot cross bun sign. All volumetric analysis results are reported as a % of total intracranial volume.

##### Patients with MSA-C compared to healthy controls

The volume of the cerebellum in patients with MSA-C (5.64 ± 0.60%) was significantly reduced compared to healthy controls (7.53 ± 1.15%); *p* < 0.001. The volume of the brainstem in patients with MSA-C (0.72 ± 0.22%) was significantly reduced compared to the healthy controls (1.25 ± 0.19%); *p* < 0.001.

##### Patients with MSA-C compared to patients with SAOA

There was no significant difference in the cerebellar volumes between patients with MSA-C and patients with SAOA (5.64 ± 0.60%) versus (4.97 ± 1.12%). However, the patients with MSA-C (0.72 ± 0.22) had a significantly reduced brainstem volume compared to the patients with SAOA (1.16 ± 0.18%) *p* < 0.001.

##### MR spectroscopy and volumetry results in the context of the presence or absence of a hot cross bun sign

Of the 20 patients diagnosed with probable MSA-C on follow up, 5 presented with no discernible hot cross bun sign on initial MR imaging. Those 5 patients had higher NAA/Cr ratios in the cerebellar hemisphere compared to those with a hot cross bun sign; (NAA/Cr 0.97 versus 0.68), but no different NAA/Cr ratios in the vermis (NAA/Cr 0.70 without vs 0.67 in those with the hot cross bun sign).

The difference in brainstem volumes of those patients with MSA-C with and those without hot cross bun was significant; 0.63% TICV versus 0.96% TICV (*p* = < 0.001).

## Discussion

The primary aim of this study was to assess the differences on ^1^H-MR spectroscopy and volumetric analysis of structural MR images between patients with MSA-C, SAOA and healthy control subjects.

^1^H-MR spectroscopy is a non-invasive imaging technique that is easily applicable in the clinical setting and has been shown to be reliable and useful when imaging posterior cranial fossa structures [19–23]. We have shown a significant reduction in NAA/Cr in the cerebellar vermis in patients with MSA-C compared to age- and sex-matched patients with SAOA. It is important to recognise that matching for age represents a selection bias. However this reflects what faces the neurologist in clinic, as by definition patients are only categorised as having SAOA after elimination of all identifiable causes. The significant reduction of NAA/Cr and Cho/Cr from the cerebellum in patients with MSA-C compared to healthy control subjects is in keeping with previous studies [22, 23]. Terakawa et al. compared patients with MSA-C and patients with “cerebellar cortical atrophy” (another term for SAOA) and were unable to distinguish between the disease entities using spectra with the voxel placed on both the cerebellar hemisphere and vermis [24]. This difference may reflect a number of methodological differences. Firstly, the Terakawa study was conducted at 1.5 T not 3 T with a TE of 135 ms rather than 144 ms and secondly they used much smaller spectroscopy voxels, 1 × 1 × 1 cm versus 1 × 2 × 2 cm in this study. The number of patients included was smaller, 16 patients with MSA-C and 7 patients with “cerebellar cortical atrophy” by comparison to our study of 20 patients with MSA-C and 20 patients with SAOA.

We found a significant difference in NAA/Cr between patients with MSA-C compared to patients with SAOA despite similar cerebellar volumes between the 2 groups. This may suggest that the neuronal integrity of patients with SAOA is maintained in keeping with a less aggressive disease progression compared to patients with MSA-C. Neuropathologically in patients with MSA-C there is myelin loss and neurodegeneration, associated with α-synuclein accumulation in oligodendrocytes. But the underlying pathological mechanisms are poorly understood; nor is it clear what the histological correlates of these MR spectroscopic changes are at present.

^1^H-MR spectroscopy studies into the pons, midbrain and medulla in patients with MSA-C have highlighted the value of measuring NAA/Cr and Cho/Cr in diagnosing MSA-C [25, 26]. Whilst the reproducibility of spectra obtained from the cerebellar vermis and hemisphere has been shown to be good; [27] the reproducibility of spectra obtained from the brainstem has been shown to be poor [28].

The results from this study showed a significant reduction in the cerebellar volume in patients with MSA-C compared to healthy controls. There was no significant difference in the cerebellar volume of patients with MSA-C and patients with SAOA. Cerebellar volume alone cannot therefore be used to distinguish patients with MSA-C from those with SAOA.

The brainstem volume in patients with MSA-C was significantly reduced compared to both patients with SAOA matched for age and sex. The reduction in brainstem volume demonstrated here is in keeping with previous studies. Burk et al. also showed a significant reduction in brainstem volume using three dimensional volumetric analysis compared to both controls and patients with idiopathic ataxia [29].

Our results showed a significant difference in brainstem volume between MSA-C patients with and without a hot cross bun sign. We also showed that the hot cross bun sign appears to arise after a reduced NAA/Cr ratio from the vermis appears to be present in some cases and has not been shown to predate the ^1^H-MR spectroscopy changes in any patients with MSA-C. The implication is that abnormal ^1^H-MR spectroscopy of the vermis may predate the brainstem atrophy and this may be a better early diagnostic MR biomarker, which may prove useful for future treatment trials. This finding though statistically significant, is based upon a small cohort that is an important consideration. Those patients without a hot cross bun sign had higher NAA/Cr ratios in the cerebellar hemisphere compared to those with a hot cross bun sign but as we have pointed out the numbers are small and so we are currently evaluating this in a larger population. Thus it is the fall in vermian NAA/Cr level that we suggest is diagnostically useful in early disease, as in those with abnormal ^1^H-MR spectroscopy from the cerebellar hemisphere also had a hot cross bun sign. The fall in vermian NAA/Cr is significantly lower in MSA-C patients when compared to SAOA patients, hence the importance of its diagnostic utility. Given that MSA-C was the 4th commonest cause (11%) of sporadic ataxias in our large cohort of patients (1500) with progressive ataxia, such diagnostic utility is a useful clinical tool [30].

The major limitations of this study are that it is retrospective and a larger sample size is required. As noted above this is particularly needed to evaluate the possible finding of early spectroscopy findings which appeared to predate brainstem atrophy in some cases. Obtaining good quality ^1^H-MR spectroscopy and volumetric imaging in patients with ataxia can be challenging. Both ^1^H-MR spectroscopy and volumetric imaging in clinical practice can be compromised by movement artefact. ^1^H-MR spectroscopy requires experience technicians to obtain spectroscopy from consistent anatomical sites to permit direct comparison.

In summary this study demonstrated that the NAA/Cr produced from ^1^H-MR spectroscopy of the cerebellum from voxels placed over the cerebellar vermis and hemisphere may be useful in distinguishing patients with MSA-C from patients with SAOA at presentation. In our clinical practice the possibility of MSA-C is raised by the neuroradiologist in a patient with a new diagnosis of adult onset ataxia either because of the presence of a hot cross bun sign or if ^1^H-MR spectroscopy shows profoundly reduced NAA/Cr within the vermis in the context of patient with a relatively short history of symptoms. This however indicates a need for a prospective study.

## Additional file

Additional file 1: **Appendix 1.** Criteria for the diagnosis of probable MSA. (DOCX 14 kb)

## Abbreviations

^1^H-MR: proton MR
ANOVA: Analysis of variance
CHESS: Chemical shift selective imaging pulse technique
Cho: Choline
cm: centimetre
Cr: Creatine
DRPLA: Dentatorubral–pallidoluysian atrophy
FA: Friedreich’s ataxia
FSL: FMRIB’s Software Library
HSD: Honest Significant Difference
MELAS: Mitochondrial encephalomyopathy, lactic acidosis, and stroke-like episodes
MERRF: Myoclonic epilepsy with ragged red fibers
mm: millimetre
ms: millisecond
MSA-C: Multiple system atrophy- cerebellar type
NAA: N-acetylaspartate
NARP: Neuropathy, ataxia, and retinitis pigmentosa
NSA: Number of signal averages
POLG: mitochondrial DNA polymerase gamma
PRESS: Point-resolved spectroscopy
SAOA: Sporadic adult-onset ataxia
SCA: Spinocerebellar atrophy
SIENAX: Structural Image Evaluation using Normalization of Atrophy cross-sectional method
SPG7: Spastic paraplegia 7
SPSS: Statistical Package for Social Sciences
T: Tesla
TE: Time to echo
TICV: Total intracranial volume
TR: repetition time
