# Supplementary material for: Is 1H-MR spectroscopy useful as a diagnostic aid in MSA-C?
Source: Cerebellum Ataxias. 2019 Jul 5;6:7. doi: 10.1186/s40673-019-0099-0 (PMC6612153; doi:10.1186/s40673-019-0099-0)
Supplement: Supplementary file 1 — Appendix 1. Criteria for the diagnosis of probable MSA. (DOCX 14 kb) [file 40673_2019_99_MOESM1_ESM.docx]

Additional file 1

Appendix 1: CRITERIA FOR THE DIAGNOSIS OF PROBABLE MSA

A sporadic, progressive, adult (>30 y)–onset disease characterized by
● Autonomic failure involving urinary incontinence (inability to control the release of urine from the bladder, with erectile dysfunction in males) or an orthostatic decrease of blood pressure within 3 min of standing by at least 30 mm Hg systolic or 15 mm Hg diastolic *and*● Poorly levodopa-responsive parkinsonism (bradykinesia with rigidity, tremor, or postural instability) *or*● A cerebellar syndrome (gait ataxia with cerebellar dysarthria, limb ataxia, or cerebellar oculomotor dysfunction)

CRITERIA FOR POSSIBLE MSA

A sporadic, progressive, adult (>30 y)–onset disease characterized by

| ● Parkinsonism (bradykinesia with rigidity, tremor, or postural instability) *or* |
| --- |
| ● A cerebellar syndrome (gait ataxia with cerebellar dysarthria, limb ataxia, or cerebellar oculomotor dysfunction)*and* |
| ● At least one feature suggesting autonomic dysfunction (otherwise unexplained urinary urgency, frequency or incomplete bladder |
| ● At least one of the additional features shown below |

ADDITIONAL FEATURES OF POSSIBLE MSA-C

| Possible MSA-P or MSA-C |
| --- |
| ● Babinski sign with hyperreflexia |
| ● Stridor |
|  |
|  |
| Possible MSA-C |
| ● Parkinsonism (bradykinesia and rigidity) |
| ● Atrophy on MRI of putamen, middle cerebellar peduncle, or pons |
| ● Hypometabolism on FDG-PET in putamen |
| ● Presynaptic nigrostriatal dopaminergic denervation on SPECT or PET  Modified from Gilman et al Second consensus statement on the diagnosis of multiple system atrophy. Neurology 2008 Aug 26; 71(9): 670–676. |
